# Sugar-Sweetened Beverages and Artificially Sweetened Beverages Consumption and the Risk of Nonalcoholic Fatty Liver (NAFLD) and Nonalcoholic Steatohepatitis (NASH)

**DOI:** 10.3390/nu15183997

**Published:** 2023-09-15

**Authors:** Tung-Sung Tseng, Wei-Ting Lin, Peng-Sheng Ting, Chiung-Kuei Huang, Po-Hung Chen, Gabrielle V. Gonzalez, Hui-Yi Lin

**Affiliations:** 1 Behavior and Community Health Sciences Program, School of Public Health, Louisiana State University Health Sciences Center, New Orleans, LA 70112, USA; ggonz4@lsuhsc.edu; 2 Social, Behavioral, and Population Sciences, Tulane University School of Public Health and Tropical Medicine, 1440 Canal Street, New Orleans, LA 70112, USA; wtlin0123@gmail.com; 3 Division of Gastroenterology and Hepatology, Tulane University School of Medicine, New Orleans, LA 70112, USA; pting1@tulane.edu; 4 Department of Pathology and Laboratory Medicine, Tulane University School of Medicine, New Orleans, LA 70112, USA; chuang17@tulane.edu; 5 Division of Gastroenterology and Hepatology, Johns Hopkins University School of Medicine, 1830 East Monument Street, 4th Floor, Baltimore, MD 21287, USA; pchen37@jhmi.edu; 6 Biostatistics Program, School of Public Health, Louisiana State University Health Sciences Center, New Orleans, LA 70112, USA; hlin1@lsuhsc.edu

**Keywords:** sugar-sweetened beverage (SSBs), artificially sweetened beverages (ASBs), nonalcoholic fatty liver disease (NAFLD), nonalcoholic steatohepatitis (NASH), NHANES

## Abstract

Nonalcoholic fatty liver disease (NAFLD) and nonalcoholic steatohepatitis (NASH) are fast becoming the most common chronic liver disease and are often preventable with healthy dietary habits and weight management. Sugar-sweetened beverage (SSB) consumption is associated with obesity and NAFLD. However, the impact of different types of SSBs, including artificially sweetened beverages (ASBs), is not clear after controlling for total sugar intake and total caloric intake. The aim of this study was to examine the association between the consumption of different SSBs and the risk of NAFLD and NASH in US adults. The representativeness of 3739 US adults aged ≥20 years old who had completed 24 h dietary recall interviews and measurements, including dietary, SSBs, smoking, physical activity, and liver stiffness measurements, were selected from the National Health and Nutrition Examination Survey 2017–2020 surveys. Chi-square tests, t-tests, and weighted logistic regression models were utilized for analyses. The prevalence of NASH was 20.5%, and that of NAFLD (defined without NASH) was 32.7% of US. adults. We observed a higher prevalence of NASH/NAFLD in men, Mexican-Americans, individuals with sugar intake from SSBs, light–moderate alcohol use, lower physical activity levels, higher energy intake, obesity, and medical comorbidities. Heavy sugar consumption through SSBs was significantly associated with NAFLD (aOR = 1.60, 95% CI = 1.05–2.45). In addition, the intake of ASBs only (compared to the non-SSB category) was significantly associated with NAFLD (aOR = 1.78, 95% CI = 1.04–3.05), after adjusting for demographic, risk behaviors, and body mass index. A higher sugar intake from SSBs and exclusive ASB intake are both associated with the risk of NAFLD.

## 1. Introduction

The prevalence of nonalcoholic fatty liver disease (NAFLD) and nonalcoholic steatohepatitis (NASH) has been increasing, with global prevalences of about 25% and 3–5%, respectively [1,2,3,4]. NAFLD is a chronic liver disorder that is associated with numerous metabolic disorders, such as obesity, insulin resistance, and dyslipidemia, and it is considered to be a leading cause of liver-related morbidity and mortality worldwide [4,5]. The strong association of NAFLD with the abovementioned metabolic risk factors has even prompted a trend to rename the condition altogether as metabolic dysfunction-associated steatotic liver disease (MASLD) [6]. NASH is a more severe subcategory of NAFLD that is characterized by inflammation, hepatocyte ballooning, and fibrosis, in addition to hepatic steatosis [7]. The difference between NASH and NAFLD is that NASH and NAFLD have different rates of progression for fibrosis. These findings correspond to one stage of progression over 14.3 years for patients with NAFLD (95% CI, 9.1–50.0 y) and 7.1 years for patients with NASH (95% CI, 4.8–14.3 y) [8]. NAFLD and NASH have become a significant public health concern. The underlying pathophysiology of NAFLD and NASH is complex and multifactorial, involving various genetic, metabolic, and environmental factors [4]. Several factors have been identified as potential risk factors for the development and progression of NAFLD and NASH. These include lifestyle factors, such as diet and physical activity, as well as genetic and environmental factors [5]. Individuals are more susceptible to NAFLD or NASH if they have obesity, type 2 diabetes, and high levels of triglycerides in the blood [1].

Obesity and metabolic syndrome are two of the most important risk factors for NAFLD and NASH [9,10]. The association between obesity and NAFLD/NASH is thought to be mediated by several mechanisms. One of the most important mechanisms is the effect of obesity on insulin resistance. Excess adipose tissue can lead to insulin resistance, which, in turn, can promote the development of NAFLD and NASH [9]. In addition, obesity is associated with chronic low-grade inflammation, which can also contribute to the development of NAFLD and NASH [10]. Dietary factors have been identified as one of the primary modifiable risk factors for NAFLD and NASH. A diet high in calories, saturated and trans fats, and refined carbohydrates has been associated with an increased risk of NAFLD and NASH [11]. Gaining weight by as little as 7 to 11 pounds and consuming diets high in sugar or calories can predict NAFLD [11]. This is because a high intake of these foods results in increased fat accumulation in the liver, insulin resistance, and inflammation [12]. Several mechanisms have been proposed to explain the association between diet and NAFLD/NASH [11,12,13,14]. One of the most important mechanisms is the effect of diet on insulin resistance. A Western diet containing high quantities of refined carbohydrates and saturated fats can lead to insulin resistance, which, in turn, can promote the development of NAFLD and NASH [11,12]. Furthermore, endothelial cell dysfunction due to obesity and binge eating is another mechanism leading to NAFLD [15]. One of the major modifiable risk factors for NAFLD and NASH is the consumption of sugar-sweetened beverages (SSBs) and artificially sweetened beverages (ASBs), because they are a rapidly consumed source of calories without leading to satiety [16,17,18,19].

The consumption of SSBs and ASBs has increased significantly in the past few decades. SSBs and ASBs are widely consumed worldwide, with the global market for carbonated soft drinks alone estimated at $392.6 billion in 2020 [16,20]. SSBs are defined as beverages that contain added caloric sweeteners, such as sucrose or high fructose corn syrup, while ASBs are beverages that contain non-nutritive artificial sweeteners, such as aspartame, saccharin, or sucralose [16,18,19,21]. The excessive consumption of SSBs has been linked to the development of obesity, type 2 diabetes, cardiovascular disease, and NAFLD [16,21]. Evidence is limited on the association between NAFLD and consumption of ASBs, but ASBs have been previously linked to insulin resistance and inflammation, which could, subsequently, increase one’s risk for NAFLD [16,18,22]. One study found that a diet low in free sugar from drinks and food results in improved hepatic steatosis in adolescent boys [14]. In particular, the excess intake of sugar and caramel coloring can increase insulin resistance and liver inflammation, which would raise one’s risk for NAFLD [21]. Another systematic review and meta-analysis of seven observational studies reported that the connection between the consumption of ASBs and NAFLD is still vague due to a lack of clinical studies on this subject [18]. One study also showed that ASBs were associated with liver disease in mice, and sucralose ingestion was shown to increase the expression of the efflux transporter P-glycoprotein (P-gp) and two cytochrome P-450 (CYP) isozymes in the intestine [23]. The effect of sucralose on first-pass drug metabolism in humans, however, has not yet been determined [23]. Furthermore, some studies have reported conflicting results, with no significant association between ASB consumption and NAFLD or NASH [18,22,24,25].

To date, the impact of sugar intake from SSBs and different types of SSBs, including regular soda and artificially sweetened beverages (ASBs), on the risk of NAFLD and NASH in population-based research is not clear after controlling for total sugar intake, total caloric intake, and BMI status. Given the high prevalence of NAFLD and NASH and the potential role of SSB and ASB consumption in their development, it is important to further investigate this association. The aim of this manuscript is to examine the association between ASBs, sugar intake from SSBs, and the risk of NAFLD and NASH.

## 2. Methods and Materials

### 2.1. Study Population

A total of 3739 adults, a nationally representative sample of the United States, were selected from the National Health and Nutrition Examination Survey (NHANES) during the 2017–March 2020 cycle dataset, which is a combined dataset that includes the 2017–2018 cycle and partial 2019–2020 data due to uncompleted data collected for the NHANES 2019–2020 cycle before COVID-19 pandemic. The survey was administered by the Centers for Disease Control and Prevention (CDC) every 2 years [26]. Eligible study subjects were selected based on the following steps. First, NHANES participants aged above 20 years old who participated in liver ultrasound transient elastography and 24 dietary recall interviews and had medical measurements, including alanine aminotransferase (ALT), aspartate aminotransferase (AST), and BMI, were selected for this study. Then, we excluded individuals who were pregnant, on a special diet, had missing data on average alcoholic drink intake per day, had more than 7 alcoholic drinks (for females) or more than 14 alcoholic drinks (for males) weekly, or have ever had 4 or more alcoholic drinks every day, reported a liver condition, including liver fibrosis, liver cirrhosis, viral hepatitis, autoimmune hepatitis, and other liver disease, or had liver cancer. We excluded population with reported diagnosis of liver conditions, including liver fibrosis and cirrhosis, because these population may have changed their health behavior to accommodate a diagnosis of liver diseases. For data quality control, study subjects who had stiffness interquartile range (IQRe) ≥ 35% were further excluded from the current study [23]. This project was reviewed and approved by the National Center for Health Statistics (NCHS) Research Ethics Review Board (ERB). All participants have signed a written informed consent document before data collection was conducted [27].

### 2.2. Data on Sweetened Soda and ASB Consumption and Dietary Patterns

Two days of 24 h recall interviews were conducted to obtain detailed food item and food component information. The first recall interview was conducted in-person in the Mobile Examination Center (MEC). A phone follow-up interview was conducted 3 to 10 days later. Each type of SSBs, including soda, fruit drinks, sweetened tea and coffee, and sport and energy drinks, were identified according to the U.S. Department of Agriculture (USDA) food codes. An average of sugar intake from SSBs and from each type of SSBs were also estimated. We set the cutoff point of sugar intake from SSBs at 36 g for men and 25 g for women, according to the American Heart Association (AHA) recommendation for the amount of added sugar intake per day [28]. In order to explore the association between the type of SSB intake and risk of having NAFLD and NASH, we further classified our study subjects into non-SSB consumers, non-soda intake, ASB intake only, regular soda intake only, and multiple-types-of-SSB consumers. Individuals who consumed soda with non-caloric artificial sweetener were only defined as ASB intake only. Because a small size of soda consumers intake both ASBs and regular soda, this population was combined with the regular soda intake only group. Individuals who consumed two or more types of SSBs or ASBs with any type of SSBs were defined as multiple-SSB consumers.

### 2.3. Assessment of NAFLD and NASH

Eligible vibration-controlled transient elastography (VCTE) participants in the 2017–2020 cycle were above 12 years old who were not pregnant, able to lie down on the exam table, without an implanted electronic medical device, or wearing a bandage on the side where measurements would be taken. VCTE was conducted to provide a controlled attenuation parameter (CAP), which estimates hepatic steatosis [29]. Individuals who had CAP < 248 were defined as having no fatty liver disease. The definition we used to classify non-alcoholic fatty liver disease (NAFLD) and non-alcoholic steatohepatitis (NASH) is CAP > 248 and alanine aminotransferase (ALT) < 25 U/L and aspartate aminotransferase (AST) < 25 U/L, and CAP > 248 and ALT > 25 U/L or AST > 25 U/L, respectively [30]. In this study, NAFLD was defined as simple steatosis without NASH.

### 2.4. Covariates

Demographic questionnaires were used to collect data on age, gender, race, and the ratio of family income to poverty (PIR) by well-trained interviewers using a computer-assisted personal interview (CAPI) system. A lifestyle pattern survey, including questions on cigarette and alcohol use, physical activity, and personal medical conditions, was administered using questionnaires at the MEC. Individuals who did not smoke at least 100 cigarettes in life (SMQ020) were defined as non-smokers. According to the question “Do you now smoke cigarettes? (SMQ040)”, former and current smokers were identified. In the alcohol use section, “Ever had a drink of any kind of alcohol (ALQ111)” and “How often drink alcoholic beverages in the past 12 months (ALQ121)” were used to recognize non-alcohol drinkers. The frequency of alcoholic beverage intake per week was calculated based on the “Average alcoholic drinks in a day during the past 12 months (ALQ130)” and “How often drank alcoholic beverages in the past 12 months (ALQ121)”. Women who consumed ≤14 drinks and men who consumed ≤21 drinks per week, and who never had >4 drinks every day (ALQ151), were defined as light-to-moderate alcohol drinkers [31]. The physical activity questionnaire was used to collect how many days in a week (and how much time in a day) are spent performing vigorous and moderate recreational activities, respectively. According to the questions (PAQ650, PAQ655, and PAQ660 for vigorous activities, and PAQ665, PAQ670, and PAQ675 for moderate activities), how much time spent performing vigorous and moderate recreational activities in a week was further estimated, respectively. Physical inactivity was defined as individuals having less than 150 min of moderate-intensity exercise or less than 75 min of vigorous-intensity exercise during leisure time per week [32]. Personal medical conditions were considered if participants were diagnosed with asthma, diabetes, fatty liver, chronic obstructive pulmonary disease (COPD), arthritis, hypertension, congestive heart failure, heart attack, weak/failing kidneys, angina, thyroid problem, or cancer/malignancy. Data on total energy, total sugar, total fat, total caffeine, and total alcohol consumption were estimated from each food component by NHANES analysts based on two days of 24 h recall interviews, which are available in the total nutrient intake dataset [26]. An average consumption of total energy, total sugar, total fat, total caffeine, and total alcohol were calculated and considered as potential confounders in this study.

### 2.5. BMI and Clinical Measurements

Body measurements, including BMI, were collected by trained technicians in the MEC. According to the CDC recommendation for adults, overweight and obese were classified based on BMI ranging from 25 to 29.9 and ≥30 kg/m^2^, respectively [33]. VCTE measurements were conducted in the MEC using the FibroScan^®^ model 502 V2 Touch equipped with a medium (M) or extra-large (XL) wand (probe) to obtain information on CAP and liver stiffness measurements (LSMs). A detailed description of the protocol and procedure is available on NHANES website [29]. Data on ALT, AST, and serum creatinine are available in the standard biochemistry profile. All methods were measured on the Roche Cobas 6000 (c501 module) analyzer. The detailed description of laboratory methodologies and operating procedures was described in laboratory method files [34]. Three parameters, including serum creatinine, age, and gender, were used to calculate estimated glomerular filtration rate (eGFR) levels [35].

eGFR(mL/min/1.73 m2)=142×min⁡(Scr/κ, 1)^α×max (Scr/κ, 1)^(−1.200)×(0.9938)^age×1.012 [if female]

*SCr* (standardized serum creatinine) = mg/dL
*κ* = 0.7 (females) or 0.9 (males)
*α* = −0.241 (females) or −0.302 (males)
*min* = the minimum of *SCr/κ* or 1
*max* = the maximum of *SCr/κ* or 1
*age* = years

### 2.6. Statistical Analysis

Due to the complex sampling design, an appropriate sampling weight was selected and used in all analyses. Data management and all statistical analyses were performed using Stata v17 (StataCorp LLC, College Station, TX, USA) under survey modules. Description results were presented using percentages under chi-square tests and means ± standard errors under simple linear regression models for categorical variables and continuous variables, respectively. Multinomial logistic regression models were used to evaluate the association between the type of soda consumption and the risk of having NAFLD and NASH. Demographic information, including age, gender, race, and PIR, were considered as potential confounders in Model 1. Then, variables in Model 1 and lifestyle patterns, such as smoking, alcohol drinking, physical activity, and personal medical conditions, and dietary factors, such as total energy, total sugar, and total fat, were adjusted in Model 2. Model 3 includes variables in Model 1 and Model 2 and is additionally adjusted for BMI.

## 3. Results

Table 1 presents the sampling-weight-adjusted distribution of demographic factors and NAFLD/NASH status. The prevalence of NASH was 20.5%, and NAFLD was 32.7% in U.S. adults. People with NAFLD/NASH are older than those with a normal liver status (48.4 ± 0.9, 53.6 ± 1.1 vs. 43.9 ± 0.9). The data showed a higher prevalence of NASH in men and Mexican-Americans, and a higher prevalence of NAFLD in female Mexican-Americans (all *p*’s ≤ 0.002). NAFLD/NASH prevalence is similar across the levels of different ratios of family income to poverty.

The sampling-weight-adjusted distribution of lifestyle, dietary patterns, and SSB-related factors, and biomedical examination among NAFLD/NASH status were demonstrated in Table 2. Alcohol use, physical activity level, intake, obesity, and medical comorbidities were significantly associated with NAFLD/NASH status (*p* < 0.001). The lowest prevalence of light-to-moderate alcohol use was observed in individuals with NAFLD (80.8% in normal, 73.5% in NAFLD, and 80.9% in NASH, *p* = 0.003). About 68.0% and 60.0% of individuals with NAFLD and NASH reported low physical activity, which was higher than the normal population (51.7%) (*p* < 0.001). The highest prevalence of medical conditions was found in individuals with NAFLD (48.3% in normal, 68.0% in NAFLD, and 58.2% in NASH, *p* < 0.001). Daily dietary patterns, including total energy, total sugar, total caffeine, and total alcohol intake, were associated with NAFLD/NASH status (all *p*’s <0.05). Individuals with NASH had higher total energy (2191 ± 43 vs. 2002 ± 31), higher total sugar (109 ± 4.5 vs. 99 ± 1.7), higher total caffeine (89.6 ± 2.2 vs. 83.2 ± 1.7), and higher total alcohol intake (8.8 ± 1.2 vs. 6.3 ± 0.4) per day than individuals with a normal liver status. A higher amount of sugar intake from SSBs was associated with NAFLD/NASH status (*p* < 0.001). Individuals with NASH and NAFLD had higher sugar intake from total SSBs (40.7 ± 2.9, 40.3 ± 2.9 vs. 35.4 ± 1.3). Regarding SSB intake, individuals who were NASH or NAFLD had higher SSB intake than individuals with a normal liver status (78.1%; 74.1% vs. 70.1%, respectively). Although the type of SSB intake is not observed to be significantly statistically different by NASH/NAFLD status, individuals with NASH or NAFLD had higher ASB intake than individuals with a normal liver status (11.4%; 13.0% vs. 7.3%, respectively). Except for eGFR, all clinical examinations, including LSM, ALT, AST, and BMI, were associated with NAFLD/NASH status (all *p*’s <0.001). Individuals with NASH had higher LSM (6.5 ± 0.3 vs. 4.8 ± 0.1), higher ALT (38.5 ± 0.9 vs. 18.3 ± 0.6), and higher AST (29.0 ± 0.6 vs. 20.4 ± 0.6) than individuals with a normal liver status. In addition, there is a higher prevalence of obese individuals with NASH and NAFLD than those who were normal weight (64.5% and 49.6% vs. 14.6%, respectively).

**Figure 1:**
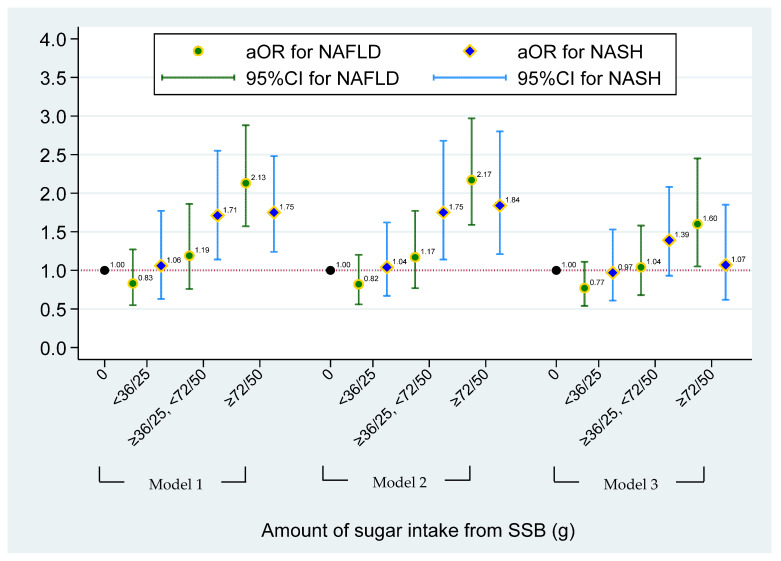
The effect of sugar intake from SSBs on risk of having NAFLD/NASH based on the multinominal logistical regression model. NAFLD was defined as simple steatosis without NASH. Model 1 was adjusted for age, gender, race, and PIR. Model 2 was adjusted for covariates in Model 1 and lifestyle pattern, including status of smoking, alcohol drinking, physical activity, and medical condition; and daily dietary intake pattern, including total energy, total sugar, and total fat. Model 3 was adjusted for covariates in Model 2 and BMI status. No more than 36 g of added sugar for men and 25 g of added sugar for women was recommended by the American Heart Association (AHA) recommendation. For heavy SSB consumers, ≥72 and ≥50 g of sugar intake from SSBs for men and women were used in this study, respectively.

**Figure 2:**
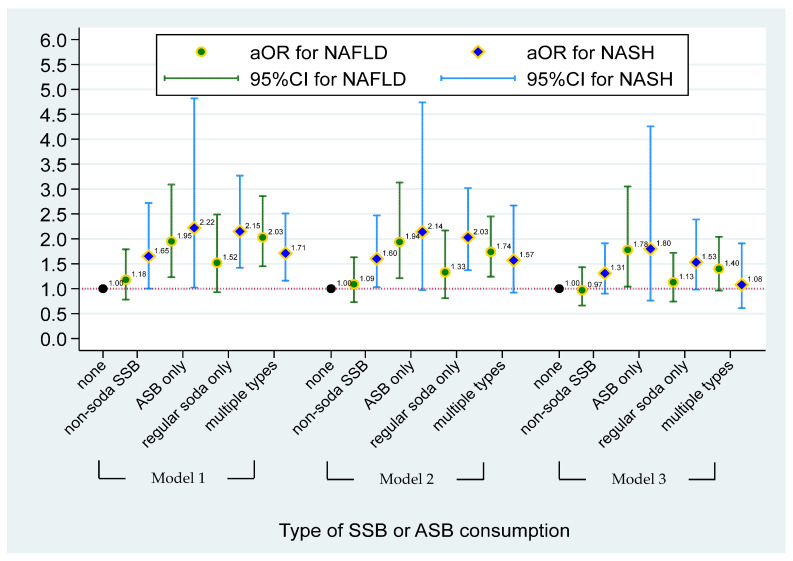
The effect of type of SSBs or ASBs consumption on risk of having NAFLD/NASH based on the multinominal logistical regression model. NAFLD was defined as simple steatosis without NASH. Model 1 was adjusted for age, gender, race, and PIR. Model 2 was adjusted for covariates in Model 1 and lifestyle pattern, including status of smoking, alcohol drinking, physical activity, and medical condition; and daily dietary intake pattern, including total energy, total sugar, and total fat. Model 3 was adjusted for covariates in Model 2 and BMI status. Non-soda intake was defined based on SSBs consumer who did not intake ASBs and regular soda. ASBs-only consumers were defined as individuals who only consumed soda with no-calorie artificial sweetener. Individuals who consumed two or more types of SSBs or ASBs and with any types of SSBs were defined as multiple-SSBs consumers.

The multinomial logistic regression model between sugar intake from SSBs and NASH/NAFLD status is shown in Figure 1. Compared with non-SSB consumers, individuals who had ≥72 (male) and 50 (female) grams of sugar intake from total SSBs had a significantly higher risk of having NAFLD (aOR = 1.60, 95% CI = 1.05–2.45), after adjusting for demographic factors, risk behaviors, and BMI (Model 3). Figure 2 illustrates the effect of the type of SSB consumption on the risk of having NAFLD/NASH based on the multinomial logistic regression model. Significant higher risks of having NAFLD were found in individuals who consumed ASBs only and multiple types of SSBs when compared to non-SSB consumers before adjusting for BMI status (aOR = 1.94, 95% CI = 1.21–3.13 in ASB-only and aOR = 1.74, 95% CI = 1.24–2.45 in multiple-types-of-SSB consumers) (Model 2). A 1.60-fold and 2.03-fold higher risk of having NASH was found in individuals who consumed non-soda SSBs (95% CI = 1.21–3.13) and regular soda only (95% CI = 1.37–3.02) when compared to non-SSB consumers, respectively, after adjusting for demographic and risk behavioral factors (Model 2). However, apart from the association of ASB drinkers with NAFLD, other aforementioned associations disappeared after adjusting for BMI. After additionally adjusting for BMI status in Model 3, a higher risk of having NAFLD was found in individuals who consumed ASBs only, when compared to non-SSB consumers (aOR = 1.78, 95% CI = 1.04–3.05). (Data were also shown in Tables S1 and S2.)

## 4. Discussion

To our knowledge, this is the first investigation of the NHANES cohort assessing the role of SSBs and ASBs in NAFLD. Our major findings are (1) the dose-dependent association of sugar intake from SSBs with NAFLD and (2) the association of exclusive ASB consumption with NAFLD after adjusting for all covariates.

The association of SSBs with NAFLD is consistent with a recent systematic review and meta-analysis by Chen et al. that found an increasing risk of NAFLD with rising levels of SSB consumption, up to a 53% increased relative risk in those who drink ≥7 cups/week [17]. It is noteworthy that we found an increased risk of NASH with sugar-sweetened beverages (both non-soda and regular soda) when adjusting for demographic/socioeconomic characteristics and risk behavior (Model 2), which was mitigated after adjusting for BMI (Model 3). This suggests a possible mediation between the effects of SSBs and weight gain that results in NASH rather than a direct effect of SSBs on steatohepatitis. To be sure, Ma et al. did demonstrate a dose-dependent association between SSB intake and continuous ALT levels after controlling for covariates, including BMI, but, while this finding was statistically significant, it was a small effect clinically [24].

Individuals with NASH or NAFLD had higher SSB and ASB consumption compared with normal individuals. Types of SSBs include beverages with high levels of fructose [21]. Fructose is commonly found in fruits, fruit juices, and honey, and is typically consumed as sucrose and high-fructose corn syrup [21]. Sucrose and high-fructose corn syrup are commonly found in SSBs, and the overconsumption of sugar from SSBs is one of the major risk factors for NAFLD and NASH [36,37]. Additionally, artificial sweeteners in diet sodas are also being consumed at a fast rate in the United States. The overconsumption of artificial sweeteners can cause gut microbiome dysbiosis and release proinflammatory mediators, which can possibly increase one’s risk for NAFLD [38]. There are mixed results on the consumption of artificially sweetened beverages leading to NAFLD, but more studies are needed to support the idea that artificial sweeteners increase NAFLD prevalence [38]. An article by Emamat and colleagues hypothesize that artificial sweeteners possibly raise the prevalence of NAFLD, but further research is needed to support this theory [19].

NAFLD is a condition where fat builds up as hepatocytes in the liver, elicited by disrupting de novo lipogenesis (DNL), fatty acid β-oxidation, fatty acid uptake, and very low-density lipoprotein (VLDL) synthesis and secretion mechanisms in the liver [39,40]. It is not surprising that our study demonstrated the association of SSBs with NAFLD/NASH since the consumption of SSBs increases DNL and fatty acid uptake mechanisms. The excessive consumption of SSBs, which contain extra caloric intake, may lead to obesity with an increased BMI. Thus, SSBs are not significantly associated with NAFLD when the analysis is performed by adjusting BMI. Unexpectedly, we revealed the association between ASBs and NAFLD when those adjustments are considered for this analysis. This is a surprising finding since ASB consumption has no extra caloric content and should not alter hepatic lipogenesis. However, it has been previously reported that ASBs might cause dysbiosis [41], which may explain our findings, since the altered microbiota in glucose-treated mice has been linked to NAFLD progression [42]. On the other hand, a later study suggested that the ASB-mediated microbiome changes are different from SSB-mediated changes (glucose-treated) [25]. Thus, the altered microbiota in the ASB-consuming population may not completely explain our findings. Interestingly, this study identified the negative impacts of ASBs on the T-cell-mediated responses [25]. It has been suggested that high T-cell numbers are correlated with NAFLD progression [43], despite the fact that controversy exists [44]. If ASBs reduced T-cell-mediated responses, which may be positively associated with NAFLD progression, we should observe no or a negative correlation between ASBs and NAFLD. However, we found ASBs are significantly and positively associated with NAFLD. Given the complicated roles of T cells in NAFLD progression and the unknown mechanisms between ASBs and NAFLD progression, further studies will be needed to clarify how ASB consumption may cause the risk of developing NAFLD.

In the current study, the effect of sugar intake from SSBs and the type of SSB intake on the risk of having NASH was explained by BMI status. Therefore, we did not observe any significant contributions from the sugar intake of SSBs or type of SSB intake to the risk of having NASH when additionally adjusted for BMI status (Model 3). Consistently, the highest proportion of obesity was estimated in individuals with NASH compared to the normal population and individuals with NAFLD.

The major ingredient in soda in the United States is predominantly high-fructose corn syrup, which contains 60–65% of fructose [45,46]. Fructose is known to be more lipogenic than glucose, and individuals with NAFLD have been shown to consume more fructose and have a higher hepatic mRNA expression of fatty acid synthase on their liver biopsy than non-NAFLD controls [47,48]. While our study was not designed to investigate the differences between fructose and non-fructose sugar intake, there were higher odds of NAFLD with regular soda compared to non-soda SSB consumption (aOR 2.03 versus 1.60) prior to adjusting for BMI. Nevertheless, the fact that both regular soda and non-soda SSB consumption did not have an association with NAFLD after adjusting for BMI is consistent with a prior interventional study showing equivalent hepatic triacylglycerol concentrations on magnetic resonance imaging (MRI) results between individuals on isocaloric fructose versus glucose diets [49]. Our observation that non-soda SSB intake, like fructose-containing regular soda, similarly increases the risk of NAFLD before the adjustment for BMI suggests the effect of SSBs towards the risk for NAFLD may be mediated by weight and potentially be an energy-mediated phenomenon rather than a specific effect of fructose versus glucose. Whether or not there is any slight incremental benefit for fructose elimination from SSBs towards NAFLD in this lens may become a difficult academic question to resolve. The more pragmatic observation may simply be that SSBs in general contribute to NAFLD.

Our finding of an association between exclusive ASB consumption and NAFLD, even after adjustment of BMI status, is novel. It may be difficult to compare with other studies due to the heterogeneity of ingredients contained in ASBs. Ma et al. conducted the largest known cross-sectional study in the United States assessing this association using the Framingham cohort of 2634 patients, and, while there was an association of ASBs with NAFLD on imaging, the association was no longer statistically significant after adjusting for BMI [24]. The findings from Ma et al. suggest ASBs may have a role in replacing SSBs as long as it does not lead to weight gain. Two key differences between our study and Framingham exist: (1) we have different definitions of NAFLD—Ma et al. used computed tomography (C.T.) instead of VCTE; and (2) the Framingham cohort comprised mostly of middle-aged Americans, whereas NHANES is representative of the U.S. population in general. Internationally, studies with SSB intervention were performed as randomized trials in Denmark, with increased liver fat content observed in subjects with 6 months of sucrose SSBs, but not with aspartame-sweetened diet cola [50,51]. Our findings were the first to report an association between ASBs and fatty liver, which were not reported in US and European studies [24,50,51].

In this study, the results also showed that physical activity, total energy, total caffeine, and total alcohol intake were associated with NAFLD/NASH status, which were consistent with previous studies. Sedentary lifestyles can potentially contribute to the development of NAFLD, and an increase in daily physical activity is recommended to avoid future poor health outcomes, such as metabolic syndrome, type 2 diabetes, and NAFLD [11]. Total energy intake is another related risk factor for NAFLD. Energy restriction is a strong method with which to reduce one’s risk for NAFLD since excess calorie consumption often leads to obesity, which is a common risk factor for NAFLD and NASH [11]. Caffeine and alcohol intake are previously linked to NAFLD and NASH. A study by Yuan and colleagues found inverse relationships between coffee, caffeine, and alcohol, but power was low in the analysis [52]. Additionally, a systematic review of coffee consumption and the progression of NAFLD found that increased coffee consumption is inversely associated with the severity of hepatitis fibrosis, but further research is needed to fully understand coffee and caffeine’s role [53].

Our study has several strengths: First and foremost, it is a nationally representative sample of the United States. Second, we were able to assess hepatic fat objectively using CAP along with serum liver enzymes to accurately categorize NASH and NAFLD. Third, the dietary assessments within the NHANES questionnaire are uniquely applicable to our study question, as they can obtain detailed reports of specific sweetened beverage intake measures from questionnaires and two days of 24 h recall surveys. Lastly, this is the first finding to report an association between ASBs and fatty liver.

On the other hand, we recognize the limitations of the current study. First, our study is limited by its cross-sectional nature, limiting inference on causation. Second, while VCTE is an objective test, it is an imperfect test with the potential for sampling bias and inter-operator variation [54], though we did exclude ineffective tests (IQRe ≥ 35%). Third, the prevalence of 32.7% and 20.5% for NAFLD and NASH, respectively, are higher, because this study uses a CAP cutoff of 248 instead of 302, and AST and ALT cutoffs are 25 for both females and males, rather than 25 for females and 35 for males. Fourth, this study includes those with diagnosed NAFLD who might be asked to reduce their BMI or change their dietary intake, and, consequently, may underestimate the impact of these associations. Fifth, due to the variation of ingredients of SSBs and ASBs between different jurisdictions, our study results may not be generalizable beyond the United States. Additionally, some potential confounders were not considered in the analyses due to data limitations, such as the genetic factors related to nonalcoholic fatty liver disease progression. Lastly, individuals with a reported diagnosis of liver fibrosis and cirrhosis were excluded because we wanted to avoid the effect of changed health behavior in these population to accommodate a diagnosis of liver fibrosis and cirrhosis. However, since only 0.7% of the study population (*n* = 7 in liver fibrosis and *n* = 23 in liver cirrhosis) were excluded, the exclusion criterion would have minimal meaningful effect to our results.

## 5. Conclusions

In conclusion, SSBs were observed to have a dose-dependent effect on the risk of NAFLD, whereas ASBs had a class effect on NAFLD. Future prospective studies that stratify the ingredients of both SSBs and ASBs may further increase our understanding of the active link between sweetened beverages and NAFLD.

## Figures and Tables

**Table 1:** Distribution of demographic factors and NAFLD/NASH.

Factors		Liver Examination Status			*p* Value
	Total	Normal	NAFLD ^1^	NASH	
**Raw population ^2^**	N = 3739	N = 1636	N = 1367	N = 736	
**Survey-weighted ^3^**	100%	46.8%	32.7%	20.5%	
**Personal characteristics**					
Age, years (mean ± se)	48.0 ± 0.7	43.9 ± 0.9	53.6 ± 1.1	48.4 ± 0.9	<0.001
Gender					
male	45.7%	41.4%	28.7%	30.0%	<0.001
female	54.3%	51.4%	36.0%	12.6%	
Race					
non-Hispanic White	63.3%	47.1%	32.6%	20.3%	0.002
non-Hispanic Black	11.5%	56.2%	33.1%	10.7%	
Mexican-American	7.1%	36.2%	36.2%	27.5%	
other Hispanic	7.3%	43.9%	31.7%	24.4%	
other Race	10.8%	43.7%	30.8%	25.5%	
PIR					
below poverty	11.3%	48.4%	32.8%	18.8%	0.754
1–1.99	16.2%	42.4%	38.9%	19.7%	
2–2.99	15.6%	46.3%	31.1%	22.7%	
3–3.00	13.9%	49.0%	29.5%	21.6%	
≥4	43.1%	47.6%	32.3%	20.1%	

^1^ NAFLD was defined as simple steatosis without NASH. ^2^ Raw number of participants in this study without adjusting for sample survey design. ^3^ Results were obtained after adjusting for sample weights and complex study design. Abbreviations: PIR, Ratio of family income to poverty.

**Table 2:** The sampling-weight-adjusted distribution of lifestyle, dietary patterns, and SSB-related factors, and biomedical examination among NAFLD/NASH status.

Factors		Liver Examination Status			*p* Value
	Total	Normal	NAFLD ^1^	NASH	
**Raw population ^2^**	N = 3739	N = 1636	N = 1367	N = 736	
**Survey-weighted ^3^**	100%	46.8%	32.7%	20.5%	
**Lifestyle patterns**					
Cigarettes use					
none	63.7%	65.5%	62.1%	62.1%	0.513
former	23.0%	20.4%	25.0%	12.2%	
current	13.3%	14.1%	12.9%	25.7%	
Alcohol use					
none	21.6%	19.2%	26.5%	19.1%	0.003
light to moderate	78.4%	80.8%	73.5%	80.9%	
Physical activity (h/week)					
low	58.0%	50.2%	68.0%	60.0%	<0.001
adequate	42.0%	49.8%	32.0%	40.0%	
Medical conditions ^4^					
no	43.2%	51.7%	32.0%	41.8%	<0.001
yes	56.8%	48.3%	68.0%	58.2%	
**Daily dietary pattern, mean ± se**					
Total caloric (kcal)	2047 ± 25	2002 ± 31	2020 ± 35	2191 ± 43	<0.001
Total sugar (gm)	102 ± 1.6	99 ± 1.7	103 ± 2.5	109 ± 4.5	0.019
Total fat (gm)	84.4 ± 1.3	83.2 ± 1.7	82.9 ± 1.5	89.6 ± 2.2	0.017
Total caffeine (mg)	162 ± 5.4	151 ± 6.1	176 ± 10.1	167 ± 8.9	0.037
Total alcohol (gm)	7.1 ± 0.4	6.3 ± 0.4	7.4 ± 0.8	8.8 ± 1.2	0.030
**SSB-related elements**					
Sugar intake from total SSBs (gm), mean ± se	38.1 ± 1.4	35.4 ± 1.3	40.3 ± 2.9	40.7 ± 2.9	0.023
Sugar intake from each SSBs, mean ± se					
Soda (gm)	20.7 ± 1.2	19.1 ± 1.3	21.3 ± 1.6	23.4 ± 2.5	0.067
Fruit drinks (gm)	5.1 ± 0.4	5.0 ± 0.6	5.4 ± 0.6	4.9 ± 0.7	0.990
Sweetened tea/coffee	9.8 ± 0.9	8.6 ± 0.7	11.7 ± 1.8	9.4 ± 1.6	0.350
Sport/energy drinks	2.5 ± 0.3	2.7 ± 0.3	2.0 ± 0.5	3.0 ± 0.7	0.914
Type of SSB intake					
non-SSB intake	26.9%	29.9%	25.9%	21.9%	0.065
non-soda intake ^5^	21.7%	22.7%	19.1%	23.5%	
ASB intake only ^6^	10.5%	7.3%	13.0%	11.4%	
regular soda only	19.8%	18.6%	18.9%	24.1%	
multiple types	21.1%	20.5%	23.2%	19.1%	
**Clinical Examination**					
LSM (kPa), mean ± se	5.5 ± 0.1	4.8 ± 0.1	5.8 ± 0.2	6.5 ± 0.3	<0.001
ALT (U/L), mean ± se	21.7 ± 0.4	18.3 ± 0.6	15.8 ± 0.2	38.5 ± 0.9	<0.001
AST (U/L), mean ± se	21.1 ± 0.3	20.4 ± 0.6	17.2 ± 0.1	29.0 ± 0.6	<0.001
eGFR, mL/min/1.73 m^2^, mean ± se	96.3 ± 0.8	99.0 ± 0.9	92.0 ± 1.4	97.2 ± 1.4	0.056
BMI					
normal weight	29.3%	53.3%	13.4%	7.1%	<0.001
overweight	32.2%	32.1%	37.0%	28.4%	
obese	38.5%	14.6%	49.6%	64.5%	

^1^ NAFLD was defined as simple steatosis without NASH. ^2^ Raw number of participants in this study without adjusting for sample survey design. ^3^ Results were obtained after adjusting for sample weights and complex study design. ^4^ Medical condition includes asthma, diabetes, fatty liver, COPD, arthritis, hypertension, congestive heart failure, heart attack, weak/failing kidneys, angina, thyroid problem, and cancer/malignancy. ^5^ Individuals who consumed sweetened drinks other than ASBs and regular soda were categorized into non-soda intake. ^6^ Individuals who only consumed soda with no-calorie artificial sweetener were defined as ASB-only.

## Data Availability

Not applicable.

## Supplementary Materials

The following supporting information can be downloaded at: https://www.mdpi.com/article/10.3390/nu15183997/s1, Table S1: The effect of sugar intake from SSBs on risk of having NAFLD/NASH based on the multinominal logistical regression model; Table S2: The effect of type of SSBs or ASBs consumption on risk of having NAFLD/NASH based on the multinominal logistical regression model.
