# Supplementary material for: Sugar-Sweetened Beverages and Artificially Sweetened Beverages Consumption and the Risk of Nonalcoholic Fatty Liver (NAFLD) and Nonalcoholic Steatohepatitis (NASH)
Source: Nutrients. 2023 Sep 15;15(18):3997. doi: 10.3390/nu15183997 (PMC10534429; doi:10.3390/nu15183997)
Supplement: Supplementary file 1 [file nutrients-15-03997-s001.zip › nutrients-2568473-supplementary.pdf]

Table S1. The effect of sugar intake from SSBs on risk of having NAFLD/NASH based on the multinominal logistical regression model.

	Liver examination status											
	Model 1				Model 2				Model 3			
**Outcome**	**NAFLD vs Normal**		**NASH vs Normal**		**NAFLD vs Normal**		**NASH vs Normal**		**NAFLD vs Normal**		**NASH vs Normal**	
	aOR	95% CI	aOR	95% CI	aOR	95% CI	aOR	95% CI	aOR	95% CI	aOR	95% CI
**Amounts of sugar intake from SSB**												
none	1.0		1.0		1.0		1.0		1.0		1.0	
<36/25 g	0.83	(0.55, 1.27)	1.06	(0.63, 1.77)	0.82	(0.56, 1.20)	1.04	(0.67, 1.62)	0.77	(0.54, 1.11)	0.97	(0.61, 1.53)
≥36/25 and <72/50 g	1.19	(0.76, 1.86)	**1.71**	(1.14, 2.55)	1.17	(0.77, 1.77)	**1.75**	(1.14, 2.68)	1.04	(0.68, 1.58)	1.39	(0.93, 2.08)
≥72/50 g	**2.13**	(1.57, 2.88)	**1.75**	(1.24, 2.48)	**2.17**	(1.59, 2.97)	**1.84**	(1.21, 2.80)	**1.60**	(1.05, 2.45)	1.07	(0.62, 1.85)

NAFLD was defined as simple steatosis without NASH.
Model 1 was adjusted for age, gender, race, and PIR.
Model 2 was adjusted for covariates in Model 1 and lifestyle pattern, including status of smoking, alcohol drinking, physical activity, and medical condition, daily dietary intake pattern, including total energy, total sugar, and total fat.
Model 3 was adjusted for covariates in Model 2 and BMI status.
No more than 36 g of added sugar for men and 25 g of added sugar for women was recommended by the American Heart Association (AHA) recommendation. For heavy SSB consumers, ≥72 and ≥50 g of sugar intake from SSBs for men and women were used in this study, respectively.

Table S2. The effect of type of SSBs or ASBs consumption on risk of having NAFLD/NASH based on the multinominal logistical regression model.

	Liver examination status											
	Model 1				Model 2				Model 3			
**Outcome**	**NAFLD vs Normal**		**NASH vs Normal**		**NAFLD vs Normal**		**NASH vs Normal**		**NAFLD vs Normal**		**NASH vs Normal**	
	aOR	95% CI	aOR	95% CI	aOR	95% CI	aOR	95% CI	aOR	95% CI	aOR	95% CI
**Type of soda intake**												
non-SSB intake	1.0		1.0		1.0		1.0		1.0		1.0	
non-soda intake [1]	1.18	(0.78, 1.79)	1.65	(1.00, 2.72)	1.09	(0.73, 1.63)	**1.60**	(1.03, 2.47)	0.97	(0.66, 1.43)	1.31	(0.90, 1.91)
ASB intake only [2]	**1.95**	(1.23, 3.09)	**2.22**	(1.02, 4.82)	**1.94**	(1.21, 3.13)	2.14	(0.97, 4.74)	**1.78**	(1.04, 3.05)	1.80	(0.76, 4.26)
regular soda only	1.52	(0.93, 2.49)	**2.15**	(1.42, 3.27)	1.33	(0.81, 2.17)	**2.03**	(1.37, 3.02)	1.13	(0.74, 1.72)	1.53	(0.98, 2.39)
multiple types	**2.03**	(1.45, 2.86)	**1.71**	(1.16, 2.51)	**1.74**	(1.24, 2.45)	1.57	(0.92, 2.67)	1.40	(0.96, 2.04)	1.08	(0.61, 1.91)

NAFLD was defined as simple steatosis without NASH.

Model 1 was adjusted for age, gender, race, and PIR.

Model 2 was adjusted for covariates in Model 1 and lifestyle pattern, including status of smoking, alcohol drinking, physical activity, and medical condition, daily dietary intake pattern, including total energy, total sugar, and total fat.

Model 3 was adjusted for covariates in Model 2 and BMI status.

[1] Individuals who consumed sweetened drinks, other than ASB and regular soda were categorized into non-soda intake.

[2] Individuals who only consumed soda with no calories artificial sweetener were defined as ASB only.

Non-soda intake was defined based on SSBs consumer who did not intake ASBs and regular soda. ASBs-only consumers were defined as individuals who only consumed soda with no-calorie artificial sweetener. Individuals who consumed two or more types of SSBs or ASBs and with any types of SSBs were defined as multiple-SSBs consumers.
